# Une cause rare d’hypercorticisme: un phéochromocytome sécrétant l’ACTH (à propos d’un cas)

**DOI:** 10.11604/pamj.2024.47.88.36616

**Published:** 2024-02-26

**Authors:** Kaoutar Rifai, Fatima Toulali, Hinde Iraqi, Meryeme Ettaik, Mohamed El Hassan Gharbi

**Affiliations:** 1 Service d’Endocrinologie et Maladies Métaboliques, Centre Hospitalo-Universitaire Ibn Sina, Faculté de Médecine et de Pharmacie, Université Mohammed V Souissi, Rabat, Maroc

**Keywords:** Phéochromocytome, syndrome de Cushing paranéoplasique, ACTH dépendant, cas clinique, Pheochromocytoma, paraneoplastic Cushing´s syndrome, ACTH-dependent, case report

## Abstract

La sécrétion ectopique d´Adrenocorticotropic hormone (ACTH) par un phéochromocytome est une cause très rare du syndrome de Cushing, posant des difficultés diagnostiques et thérapeutiques. Nous rapportons le cas d´une patiente chez qui ce diagnostic a été suspecté devant un syndrome de Cushing sévère associé à une mélanodermie, une hypertension artérielle résistante à une trithérapie et un diabète déséquilibré sous insulinothérapie. Biologiquement, les dérivés méthoxylés urinaires, le cortisol libre urinaire de 24 heures ainsi que l´ACTH étaient très élevés. L'imagerie a montré une masse surrénalienne gauche de 3,5 cm. La patiente a bénéficié d´une surrénalectomie gauche après une préparation médicale avec une bonne évolution clinico-biologique. L´étude anatomopathologique a confirmé le diagnostic de phéochromocytome. Notre cas illustre l´intérêt du dosage des dérivés methoxylés devant tout syndrome de Cushing ACTH dépendant associé à une masse surrénalienne. Ceci dans le but d´assurer une prise en charge précoce et d´éviter les complications pouvant engager le pronostic vital.

## Abstract (en)

Ectopic ACTH-secreting pheochromocytoma is a very rare cause of Cushing´s syndrome, posing diagnostic and therapeutic challenges. We here report the case of a female patient with suspected severe Cushing´s syndrome associated with melanoderma, arterial hypertension resistant to triple therapy and unbalanced diabetes treated with insulin therapy. Biologically, urinary ethoxylated, 24-hour urinary free cortisol and ACTH were very high. Imaging showed a 3.5 cm left adrenal mass. The patient underwent left adrenalectomy after medical preparation, with good clinico-biological outcome. Anatomopathological examination confirmed the diagnosis of pheochromocytoma. This case study highlights the importance of measuring methoxylated derivatives in any patient with ACTH-dependent Cushing´s syndrome associated with an adrenal mass. The aim is to ensure early treatment and avoid life-threatening complications.

## Introduction

Le phéochromocytome est une tumeur neuroendocrine rare, développée à partir des cellules chromaffines de la médullo-surrénale, avec une incidence annuelle d´environ 3-8 nouveaux cas par million d´habitants. Sa prévalence chez les patients atteints d´hypertension artérielle est de 0,1-0,6% [1]. La symptomatologie clinique du phéochromocytome est très variable, donnant à ce dernier la caractéristique du « grand imitateur ». La plupart des signes cliniques sont secondaires à la sécrétion de catécholamines (adrénaline, noradrénaline et dopamine) [1]. Dans de rares cas, le phéochromocytome peut également sécréter des interleukines, de la calcitonine et de l´ACTH. La sécrétion de l´ACTH par le phéochromocytome est responsable d´un syndrome d´hypercorticisme chronique. Il s´agit d´une cause exceptionnelle du syndrome de Cushing ACTH dépendant par sécrétion ectopique d´ACTH (< 0,5% des cas) [2]. Très peu de cas de sécrétion ectopique d´ACTH par le phéochromocytome ont été rapportés dans la littérature [2]. Nous rapportons un cas rare d´une patiente âgée de 55 ans présentant un syndrome de Cushing sévère en rapport avec un phéochromocytome sécrétant l´ACTH.

## Patient et observation

**Informations du patient:** il s´agit d´une patiente âgée de 55 ans, suivie pour hypertension artérielle résistante sous trithérapie depuis 3 ans, diabète déséquilibré sous insulinothérapie, et psychose sévère sous traitement. La patiente a été admise, au service d´endocrinologie et maladies métaboliques du centre hospitalier universitaire IBN SINA de Rabat, pour prise en charge d´un syndrome de Cushing sévère.

**Figure 1:**
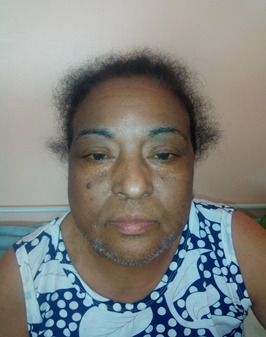
faciès Cushingoide avec mélanodermie et hirsutisme

**Résultats cliniques**: l´examen clinique a objectivé une obésité facio-tronculaire contrastant avec un aspect grêle des membres inférieures, des ecchymoses spontanées des membres et du tronc, avec une mélanodermie intéressant le visage, les mains et les pieds, le tout évoluant dans un contexte d´altération de l´état général (Figure 1).

**Chronologie**: le début de cette symptomatologie clinique remonte à 3 ans, et devant l´aggravation de la psychose et le déséquilibre glycémique, la patiente a consulté dans notre formation.

**Figure 2:**
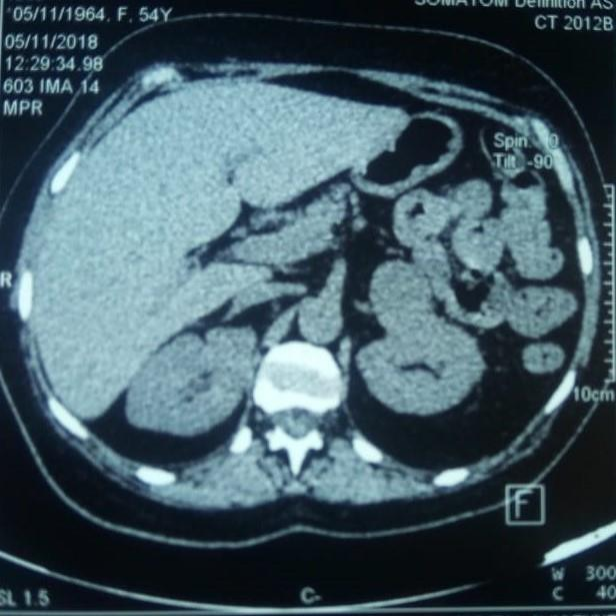
aspect scannographique de la masse surrénalienne gauche

**Démarche diagnostique**: sur le plan biologique, le diagnostic de syndrome de Cushing a été confirmé par un cortisol libre urinaire (CLU) de 24 heures très élevé à 13144 nmol/24h soit 54 fois la normale. Comme retentissement hydroélectrolytique, la patiente avait une alcalose hypokaliémiante sévère avec une kaliémie basse à 1,6 mEq/L et des réserves alcalines élevées à 50 mEq/L. Par ailleurs le bilan a objectivé un profil d´hypothyroïdie centrale avec une TSHus basse à 0,13 mUI/l et une FT4 effondrée à 0,6 ng/dl. Concernant le bilan étiologique: l´ACTH était très élevée à 182 pg/ml, l´imagerie par résonance magnétique hypothalamo-hypophysaire était normale, et le freinage fort n´a pas été réalisé à cause de l´hypokaliémie sévère. À la recherche d´un foyer tumoral avec sécrétion ectopique d´ACTH, un scanner cervico-thoraco-abdomino-pelvien a été réalisé montrant ainsi la présence d´une masse surrénalienne gauche mesurant 35 mm de grand axe (Figure 2). Devant cette masse surrénalienne, un dosage des dérivés méthoxylés urinaires de 24 heures a été demandé et qui étaient très élevés à 8 fois la normale posant ainsi le diagnostic de phéochromocytome avec sécrétion ectopique d´ACTH.

**Figure 3:**
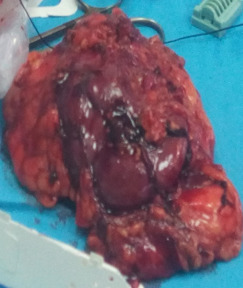
aspect macroscopique de phéochromocytome avec pièce de néphrectomie

**Intervention thérapeutique**: sur le plan thérapeutique, la patiente a bénéficié d´une supplémentation potassique par voie centrale avec une préparation médicale pendant 14 jours à base de Kétoconazole, à la dose de 600 mg par jour avec surveillance du bilan hépatique, associé à la Doxazosine à la posologie de 4mg par jour à atteindre progressivement. Par la suite la patiente a bénéficié d´une surrénalectomie avec néphrectomie gauche du fait de l´envahissement rénale de la tumeur (Figure 3). Les suites opératoires étaient simples. Et l´étude anatomopathologique a été en faveur d´un phéochromocytome gauche mesurant 3,5 cm avec un score de Pass égal à 1. Le complément immunohistochimique a confirmé le diagnostic de phéochromocytome exprimant la synaptophysine, la chromogranine A, la S100, et non pas l´ACTH.

**Figure 4:**
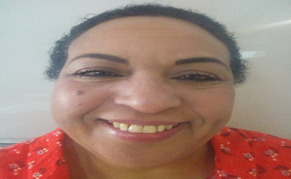
régression de la mélanodermie et de l´hirsutisme après surrénalectomie

**Suivi et résultats des interventions thérapeutiques**: après le geste opératoire, l´évolution a été marquée cliniquement par une régression spectaculaire de la mélanodermie (figure 4), une amélioration des chiffres glycémiques avec arrêt de l´insulinothérapie au bout d´une semaine, une normalisation des chiffres tensionnelles et arrêt de tout traitement antihypertenseur avec amélioration de la psychose. Sur le plan biologique, nous avons noté une normalisation du CLU et des dérivés méthoxylés et des autres paramètres hormonaux, ainsi qu´une normalisation de l´ACTH (Tableau 1) avec un suivi de 36 mois.

**Tableau 1:** évolution des paramètres biologiques après surrénalectomie gauche précédée par une préparation médicale par Doxazosine et Kétoconazole

Paramètres biologiques	En Préopératoire	A une semaine du postopératoire	A 36 mois du post-opératoire	Valeurs normales
CLU de 24 heures nmol/24 H	13144	81	85	32-243
Normétanephrines µmol/24H	18	2,45	2,30	0,4-2,1
Métanéphrines µmol/24 h	2,38	0,33	0,47	0,2-1
ACTH pg/ml	182	30	28	7-60
TSH mUI / L	0,13	2,7	3,2	0,4-4
FT4 ng/dl	0,6	0,9	0,85	0,7-1,48

**Perspectives du patient**: la patiente est très satisfaite de la bonne évolution clinico-biologique.

**Consentement éclairé**: la patiente a déclaré son consentement librement et de façon éclairée, afin de permettre la réalisation et la publication de ce manuscrit.

## Discussion

Notre cas clinique illustre une cause exceptionnelle de syndrome de Cushing secondaire à une sécrétion ectopique d´ACTH par un phéochromocytome, et qui pose des difficultés diagnostiques et thérapeutiques. Très peu de cas ont été rapportés dans la littérature. Le syndrome de Cushing a été décrit pour la première fois en 1932 par Cushing, il s´agit d´une maladie rare répondant à deux cadres physiopathologiques: a) un hypercortisolisme ACTH indépendant dans 20% des cas; b) un hypercortisolisme ACTH dépendant dans 80% des cas. Dans 90% des cas, l´ACTH est d´origine eutopique sécrétée par un adénome hypophysaire corticotrope, c´est la maladie de Cushing. Dans environ 10% des cas, l´ACTH est d´origine ectopique produite par une tumeur neuroendocrine extrahypophysaire [3] dont les plus fréquentes [4,5] sont: le cancer du poumon à petites cellules, les carcinoïdes bronchiques, le carcinome médullaire de la thyroïde, le phéochromocytome qui est encore plus rare que ces trois derniers (<5%) [2]. En effet, de rares cas ont été rapportés dans la littérature.

En 2018, Jenan et collègues ont revu une série de 58 cas rapportés dans la littérature anglaise depuis 1977 [5]. Dans cette série, il y avait une nette prédominance féminine (82 %) avec un âge moyen de 50 ans. Sur le plan clinique, la majorité des patients avaient un syndrome de Cushing sévère associé à une hypokaliémie profonde et à une hypertension artérielle résistante, comme c´était le cas pour notre patiente. Sur le plan morphologique, la plupart des patients présentaient une masse surrénalienne de grande taille avec une localisation gauche dans 62% [5]. Dans cette série, uniquement deux cas avaient une hypothyroïdie centrale [5] qui s´est corrigée après administration des anticortisoliques, et ceci a été également observé chez notre patiente. En 1979, Forman *et al*. ont proposé une série de 5 critères diagnostiques pour affirmer la présence d´un syndrome de Cushing par sécrétion ectopique d´ACTH par un phéochromocytome [4]. Ces critères ont ensuite été affinés par Dysseleer *et al*. en 1995 [2]. Ces critères sont les suivants: Un hypercorticisme biologique et clinique bien démontré, des taux élevés d´ACTH, l´évidence biochimique et radiologique d´un phéochromocytome, la disparition des symptômes liés à l´hypercorticisme et à l´excès de catécholamines après surrénalectomie unilatérale et la normalisation rapide d´ACTH après exérèse de la tumeur surrénalienne. On pourrait y ajouter un 6^e^ critère: la mise en évidence immunohistochimique de l´ACTH au sein de la tumeur médullosurrénalienne [2].

Notre patiente répondait bien aux 5 premiers critères car elle présentait un syndrome de Cushing ACTH dépendant confirmé par un CLU de 24 heures très élevé, une ACTH augmentée. Le diagnostic de phéochromocytome était posé biologiquement devant un taux élevé des dérivés méthoxylés de 24 heures avec la présence d´une masse surrénalienne gauche. L´évolution clinicobiologique spectaculaire après surrénalectomie gauche constitue un argument fort, plaidant pour un syndrome de Cushing en rapport avec une sécrétion ectopique d´ACTH par le phéochromocytome. Enfin, notre cas clinique présente une particularité immunohistochimique notamment la non expression de l´ACTH, ceci pourrait s´expliquer par l´absence d´identification de l´épitope; cette hypothèse a été proposée par Lois *et al*. [6], ou par une sécrétion ectopique de CRH qui reste la plus probable chez notre patiente vue que le taux d´ACTH n´était pas trop élevé mais ça reste à prouver par immunomarquage qui n´est pas disponible dans notre contexte.

## Conclusion

Bien que rare, le phéochromocytome sécrétant l´ACTH doit être diagnostiqué précocement pour prévenir la morbi-mortalité secondaire au tableau clinique dévastateur. Ainsi, il faut y penser et demander le dosage des dérivés methoxylés urinaires de 24 heures ou plasmatiques devant tout syndrome de Cushing ACTH dépendant associé à une masse surrénalienne afin d´assurer un diagnostic précoce et une prise en charge adéquate pour éviter la survenue de complications pouvant engager le pronostic vital.
